# Does stress mess with rodents’ heads? Influence of habitat amount and genetic factors in mandible fluctuating asymmetry in South American water rats (*Nectomys squamipes*, Sigmodontinae) from Brazilian Atlantic rainforest remnants

**DOI:** 10.1002/ece3.7557

**Published:** 2021-05-02

**Authors:** Aldo Caccavo, Hudson Lemos, Luana S. Maroja, Pablo Rodrigues Gonçalves

**Affiliations:** ^1^ Programa de Pós‐Graduação em Ciências Ambientais e Conservação PPGCiAC ‐ Instituto de Biodiversidade e Sustentabilidade NUPEM Universidade Federal do Rio de Janeiro Macaé Brazil; ^2^ Instituto de Biodiversidade e Sustentabilidade NUPEM Universidade Federal do Rio de Janeiro Macaé Brazil; ^3^ Department of Biology Williams College Williamstown MA USA; ^4^ Setor de Mastozoologia Departamento de Vertebrados Museu Nacional Universidade Federal do Rio de Janeiro Rio de Janeiro Brazil; ^5^ Museu de História Natural do Ceará Prof. Dias da Rocha Centro de Ciências da Saúde Universidade Estadual do Ceará Ceará Brazil

**Keywords:** developmental changes, habitat availability, human impacts, rodents

## Abstract

Loss of developmental stability can lead to deviations from bilateral symmetry (i.e. Fluctuating Asymmetry ‐ FA), and is thought to be caused by environmental and genetic factors associated with habitat loss and stress. Therefore, levels of FA might be a valuable tool to monitor wild populations if FA serves as an indicator of exposure to stress due to impacts of habitat loss and fragmentation. In studies examining FA and habitat fragmentation, FA levels are often explained by loss of genetic variation, though few studies have addressed FA’s use as indicator of environmental impact. Here, we investigated whether habitat loss, genetic variation, and/or inbreeding affect the developmental instability in Brazilian Atlantic forest populations of a Neotropical water rat (*Nectomys squamipes*). We sampled individuals from eight sites within Atlantic forest remnants with different amounts of available forest habitat and assessed FA levels with geometric morphometric techniques using adult mandibles. We used observed heterozygosity (H_o_) and inbreeding coefficient (F_is_), from seven microsatellite markers, as a proxy of genetic variation at individual and population levels. Populations were not significantly different for shape or size FA levels. Furthermore, interindividual variation in both shape and size FA levels and interpopulational differences in size FA levels were best explained by chance. However, habitat amount was negatively associated with both interpopulational variance and average shape FA levels. This association was stronger in populations living in areas with <28% of forest cover, which presented higher variance and higher average FA, suggesting that *Nectomys squamipes* might have a tolerance threshold to small availability of habitat. Our work is one of the first to use FA to address environmental stress caused by habitat loss in small mammal populations from a Neotropical biome. We suggest that shape FA might serve as a conservation tool to monitor human impact on natural animal populations.

## Graphical abstract

We used geometric morphometric data to asses whether fluctuating asymmetry (FA) on mandibles of Neotropical water rats (*Nectomys squamipes*) from eight sites within Brazilian Atlantic forest remnants would be related to habitat amount, genetic diversity, and/or inbreeding on individual and populational level. FA might be a valuable tool to monitor wild populations, as it might serve as an indicator of exposure to stress due to impacts of habitat loss and fragmentation. We used observed heterozygosity and inbreeding coefficient from seven microsatellite markers, as a proxy of genetic variation. We found that habitat availability rather than genetic or biological factors was negatively associated with both inter‐populational variance and average shape FA levels, which are higher in populations living in areas with <28% of habitat available. It suggests that water rats might have a tolerance threshold to small availability of habitat. We propose that shape FA might serve as a conservation tool to monitor human impact on natural animal populations.

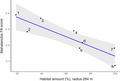

## 1 INTRODUCTION

Anthropogenic degradation of natural habitats has been one of the major causes of biodiversity decline worldwide (Haddad et al., 2015). Deforestation has reduced the amount of natural habitat available to forest‐dependent species, leaving the remaining forest cover fragmented into a series of patches with varying configurations of connectivity, size, and shape. While there is an ongoing debate on whether fragmentation “per se” is a major driver of biodiversity loss (Fahrig, 2019; Fletcher et al., 2018; Jackson & Fahrig, 2016; Püttker et al., 2020), an emerging consensus has been achieved around the negative effects of natural habitat loss on species richness in communities (Banks‐Leite et al., 2014; Fahrig, 2013; Martin, 2018; Watling et al., 2020).

The direct effects of habitat loss on biodiversity, named the Habitat Amount Hypothesis (HAH, Fahrig, 2013), states that the species richness in a given sampling site increases according to the amount of habitat in the local landscape. Aside from the habitat amount in the landscape, no other effects from the habitat patch where the sampling site is located are expected to influence species richness (Fahrig, 2013). While the HAH has been extensively tested in community studies (Fahrig, 2017, 2019; Martin, 2018; Saura, 2021), less is known about the effects of varying amounts of forest cover on stress indicators of forest‐dependent species.

Factors reducing the efficient use of energy available for growth and reproduction are sources of stress that compromise the long‐term viability of populations (Escós et al., 2000; Graham et al., 2010). Stress usually affects the development of individuals by disturbing their canalization mechanism—*that is,* the buffering against environmental variations during the developmental process leading to the formation of an optimal and stable phenotype in natural populations (Waddington, 1942)—increasing the likelihood of deviations from the optimum phenotype and giving rise to developmental instability (Klingenberg & Nijhout, 1999; Palmer, 1996). In organisms that have defined symmetric patterns, this developmental instability leads to small and random shifts from the perfect symmetry and is known as Fluctuating Asymmetry (Palmer, 1994; Tomkins & Kotiaho, 2001).

Several studies have documented a positive relationship between Fluctuating Asymmetry (FA) and stress levels in populations of many species (Anciães & Marini, 2000; Hoelzel et al., 2002; Leamy et al., 1999; McKenzie & Clarke, 1988; Pankakoski, 1985; Parsons, 1990, 1992; Sarre, 1996; Schmeller et al., 2011). Higher FA levels have been found in populations of birds, voles (Marchand et al., 2003), field mice (Maestri et al., 2015), shrews (Sánchez‐Chardi et al., 2013), and lizards (Lazić et al., 2015) living in disturbed or less suitable habitats, as well as in populations of shrews (White & Searle, 2008) and roe deer (Zachos et al., 2007) exhibiting reduced genetic variation (see reviews in Benítez et al., 2020; Klingenberg, 2015). Therefore, populations in optimal conditions tend to exhibit low FA levels, while those exposed to stressful conditions have higher levels of average FA (Shadrina & Vol'pert, 2016). These observations suggest FA as a useful indicator of stress caused by both environmental and/or genetic factors (Graham et al., 2010; Oleksyk et al., 2004).

One of the most dramatic examples of large‐scale anthropogenic environmental changes in the world's tropical forests is that of the Brazilian Atlantic forest. This forest once covered 150 million hectares along the coastal region in Brazil, but today it retains only 28% of its original cover (Rezende et al., 2018). Most of this remaining forest is highly fragmented, and 80% of its fragments are fewer than 50 hectares and are located, on average, 1440m far from each other (Ribeiro et al., 2009). Along with deforestation, Brazilian Atlantic forest is threatened by additional factors, such as the presence of invasive alien species and proximity to areas with intense human activity. As an example, the wild pig (*Sus scrofa*) competes with and reduces population sizes of several native mammals in Atlantic forest remnants (Hegel et al., 2019). In addition, proximity to humans may promote the occurrence of epizootic events in wild populations, as observed with malaria and yellow fever in non‐human primates (Buery et al., 2017; Moreno et al., 2013). Another threat is the extensive presence of domestic cats and dogs in remnant forest patches in proximity to human houses and cropland/pasture (Paschoal et al., 2018). These large domestic animals compete with the local fauna for territory, increase predation of small to medium size vertebrates, and transmit pathogens (de Almeida Curi et al., 2010; Lessa et al., 2016; Paschoal et al., 2016; Srbek‐Araujo & Chiarello, 2008).

Few studies assessed the effects of habitat loss on developmental instability in Brazilian Atlantic forest organisms. Some results indicate a negative relationship between size of forest remnants and FA, suggesting that populations in larger remnants present lower FA levels than populations in smaller forest patches (Anciães & Marini, 2000; Coda et al., 2017; Lens et al., 1999). Other results indicated a negative relationship between FA and climatic suitability, suggesting higher developmental instability in less favorable climates (Maestri et al., 2015). These studies speculated that genetic factors (e.g., inbreeding) might be involved in FA variation, although they did not directly measure genetic variation nor considered the influence of landscape factors, such as the amount of habitat, as predicted by HAH.

**FIGURE 1:**
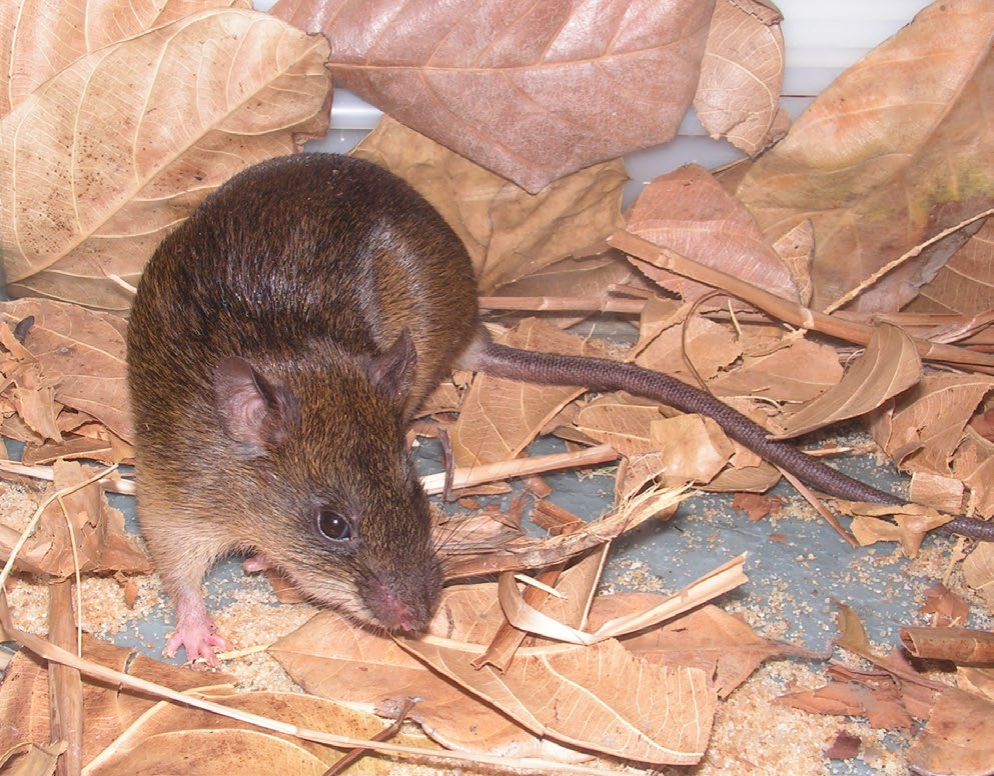
Individual of *Nectomys squamipes* captured at Restinga de Jurubatiba National Park, Rio de Janeiro, Brazil. Photograph by Pablo R. Gonçalves

In this study, we evaluated whether habitat loss and/or genetic variation affect the developmental instability of a Neotropical small mammal, the water rat *Nectomys squamipes* (Brants, 1827). This rodent (Figure 1) is widely distributed throughout remnants of Atlantic forest, especially in forests with less dense vegetation cover, small water bodies (Galliez & Fernandez, 2012; Prevedello et al., 2010), and wet soils (Ernest & Mares, 1986). In forested areas, the species preferably inhabits flooded areas and riverine habitats (Alho, 1982), being classified as a habitat specialist (Bonvicino et al., 2002). Because of its dependence on wet habitats, *Nectomys squamipes* has low dispersal capacity in dry fragmented landscapes but is able to disperse up to about 520m among forest fragments if appropriate habitats are present (Passamani & Fernandez, 2011a, 2011b; Passamani & Ribeiro, 2009; Pires et al., 2002). Water rats are also hosts to several ecto and endoparasites, some of them exotic, such as the bilharzia‐causing trematode *Schistosoma mansoni*, which consistently uses *N. squamipes* as a secondary host (D’Andrea et al., 2000; Gentile et al., 2006). Its strong dependency on forested habitats, low vagility, and vulnerability to invasive pathogens makes water rats good models to assess the effects of forest reduction and fragmentation on FA.

Here, we test whether forested habitat amount affects the levels of Fluctuating Asymmetry in *Nectomys squamipes’* mandible size and shape. We also evaluate whether FA is influenced by the heterozygosity levels and inbreeding of individuals and populations. We report no relationship between heterozygosity and FA levels, but a negative relationship between habitat amount and average mandible shape FA. Our results are important to understand the effects of reduced natural ecosystems availability on the morphological variability and viability of populations of wild mammals and to evaluate the use of these approaches in species and habitats from Neotropical biomes.

## 2 MATERIALS AND METHODS

### 2.1 Age and sexual variation

**FIGURE 2:**
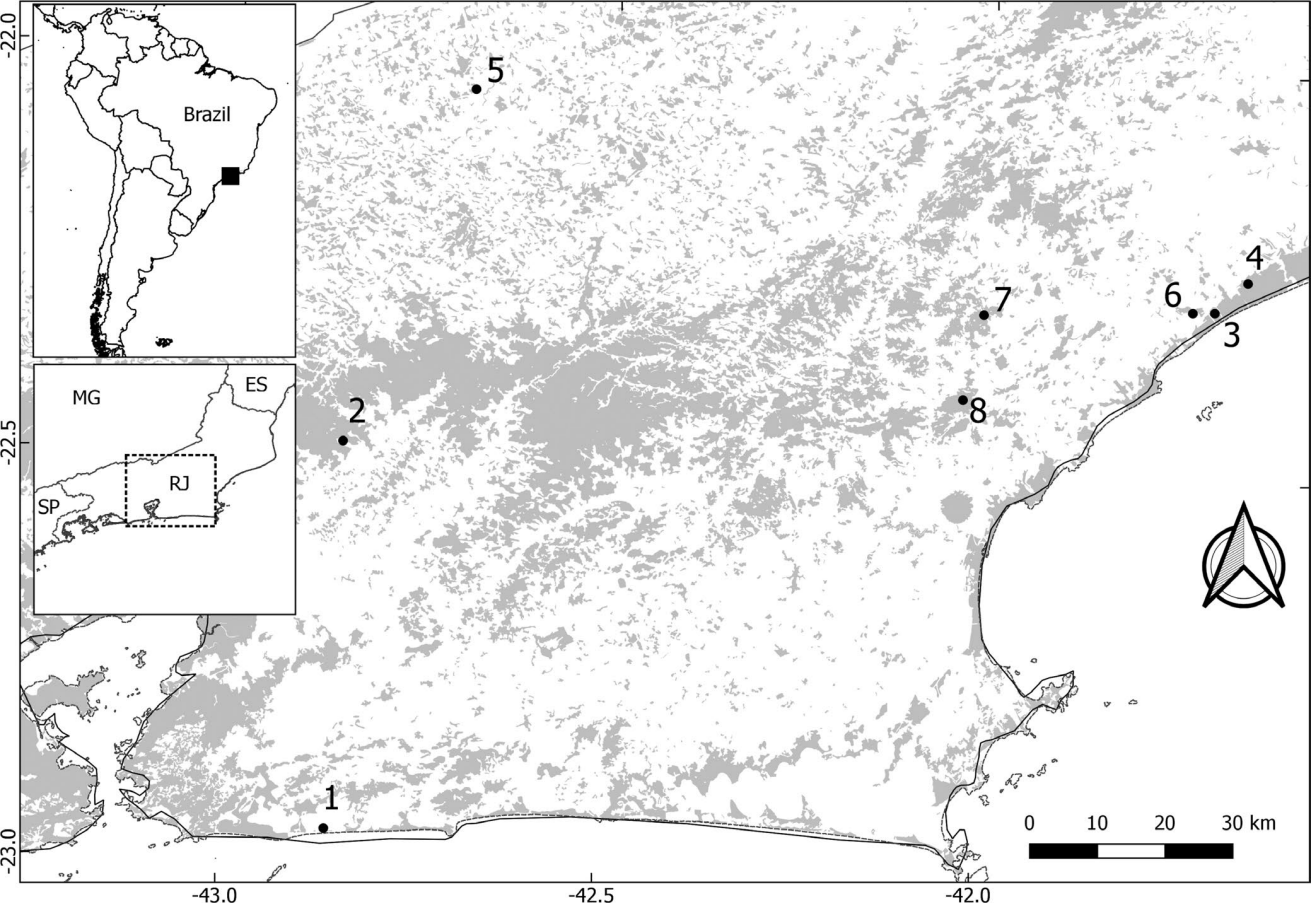
Populations samples from Atlantic forest remnants in Rio de Janeiro state. 1—Barra de Maricá; 2—Fazenda Rosimary; 3—PNRJ Lagomar (Macaé); 4—PNRJ São Lázaro (Carapebus); 5—Vale do Pamparrão; 6—Cabiúnas; 7—PNMF Atalaia; 8—ReBio União. ES: Espírito Santo state; MG: Minas Gerais state; RJ: Rio de Janeiro state; SP: São Paulo state. Gray areas represent the Atlantic forest fragments. Shapefile obtained at mapas.sosma.org.br

We examined mandibles from 87 specimens collected in eight different sites within Atlantic forest remnants in the state of Rio de Janeiro, Brazil (Figure 2, Table 1).

**TABLE 1:** Collection location, coordinates, collection date, sample size (*N*) and number of genotyped individuals (*N*_g_). * = Obtained from Almeida et al., 2005

Location	Coordinates	Date	*N*	*N*_g_
1 – Barra de Maricá, Maricá County	22°57'20.70"S; 42°50'0.00"W	1988	8	—
2 – Fazenda Rosimary, Cachoeiras de Macacu County	22°28'60.00"S; 42°51'0.00"W	2000	9	—
3 – Restinga de Jurubatiba National Park, Lagomar (Parque Nacional da Restinga de Jurubatiba, PNRJ Macaé), Macaé County	22°18'7.41"S; 42° 0'18.39"W	2014–2015	11	7
4 – Restinga de Jurubatiba National Park, Fazenda São Lázaro (PNRJ Carapebus), Carapebus County	22°15'9.14"S; 41°39'22.03"W	2007–2012	11	6
5 – Vale do Pamparrão, Sumidouro County	22°2'46.00"S; 42°41'21.00"W	2000	15	18*
6 – Cabiúnas, Macaé County	22°17'28.57"S; 41°43'40.89"W	2007–2011	11	10
7 – Municipal Natural Park Fazenda Atalaia (Parque Natural Municipal Fazenda Atalaia, PNMF Atalaia), Macaé County	22°18'7.41"S; 42° 0'18.39"W	2007–2011	13	12
8 – União Biological Reserve (Reserva Biológica União, ReBio União), Casimiro de Abreu County	22°24'26.10"S; 42° 1'44.88"W	2007	9	8*

The specimens examined are deposited in the collections of mammals of Museu Nacional (MN) and of Instituto de Biodiversidade e Sustentabilidade de Macaé (NUPEM), Universidade Federal do Rio de Janeiro (Museum accession number for each specimen is given in the Appendix). The individuals had their age class estimated using eruption and wear of the molars and, to minimize possible ontogenetic effects in FA patterns, only adults were included (i.e., individuals with all molars erupted and with signs of wear). Because studies of morphological variation in the genus *Nectomys* suggest the absence of secondary sexual dimorphism (Coutinho et al., 2013; Stein, 1988), we pooled males and females for our analyses.

### 2.2 Habitat amount

Image classification—We used Landsat satellite images recorded during the specimen collection year to calculate habitat amount for each site. We trimmed each image to the region of interest and classified habitat coverage with Landcover Signature Classification (LSC); regions with signature overlap or that could not be classified using LSC were classified using Maximum likelihood with threshold of 0.05. We then grouped the coverage categories, forming an image with a binary classification, that is, “habitat” (forests and encompassed small waterbodies) and “nonhabitat” (open areas, extensive water masses, croplands, and other anthropic land covers). For image acquisition and classification, we used the Semi‐Automatic Classification Plugin (Congedo, 2013) from the QGIS software.

To estimate the radius used to calculate habitat coverage, we calculated the scale effect of habitat amount, *that is,* the extent of the landscape in which the habitat amount best predicts the effects on the populations of *N. squamipes*. The determination of scale effect followed the procedure suggested by Melo et al. (2017). We obtained home range values for *N. squamipes* from Bergallo and Magnusson (2004) and Ernest and Mares (1986). From these home range values, we estimated the maximum dispersal distances following Bowman et al. (2002) and the possible amplitudes for the scale effect using the Jackson and Fahrig (2012) method (see Table 2). Among the values obtained, we selected four values as radius for estimates of habitat amount: 264, 562, 938, and 1426 m. We selected a wide range of biologically relevant radius (within dispersal capacity) to evaluate their potential effect in habitat amount estimates.

**TABLE 2:** Home Range and other habitat measurements of *Nectomys squamipes* used to calculate the scale effect. Bold values were used to estimate habitat amount. BS: Breeding Season; NBS: Nonbreeding Season. HR: Home range. LD: Linear distance. MDD: Maximum dispersal distance

Source		HR (m^2^)	LD (m)	MDD (m) [40 × LD]	Scale effect	
					Min (MDD 30%)	Max (MDD 50%)
Ernst and Mares (1986)	General	2,200	46.9	1,876.2	**562.8**	**938.1**
Bergallo and Magnusson (2004)	Male – BS	5,084.8	71.3	2,852.3	855.7	**1,426.2**
	Female – BS	1,260.2	35.5	1,420.0	426.0	710.0
	Male – NBS	1,829.7	42.8	1,711.0	513.3	855.5
	Female – NBS	486.7	22.1	882.5	**264.7**	441.2

For each site, we calculated the percentage of habitat amount with Landscape Ecology Statistics v. 1.9.8 (Jung, 2016) plugin for Qgis based on distance buffers from collection site coordinates using the four scale values given above (Table 3).

**TABLE 3:** Habitat amount estimates, using the selected scale effect values, for each collection site in Atlantic forest

Collection sites	264m (%)	562m (%)	938m (%)	1426m (%)
1– Barra de Maricá	18.86	16.85	14.91	11.33
2– Fazenda Rosimary	28.15	42.46	63.87	76.73
3– PNRJ Macaé	39.26	48.08	46.63	40.23
4– PNRJ Carapebus	66.25	57.71	34.76	22.01
5– Vale do Pamparrão	71.02	48.49	38.61	31.24
6– Cabiúnas	73.22	32.93	23.07	17.95
7– PNMF Atalaia	99.59	99.54	94.87	84.27
8– ReBio União	100.00	99.72	98.78	93.42

### 2.3 Digitalization and FA calculation

To assess FA levels, we focused on mandibles, a morphological system extensively used in morphological integration and FA studies (e.g., Klingenberg et al., 2003; Leamy, 1993; Leamy et al., 2015). Mandibles constitute symmetric structures with well‐known developmental basis and a small number of landmarks can represent their general shape in a geometric morphometrics context. We used FA indexes based on geometric morphometric data, applicable to museum specimens and already used in studies involving FA in small mammals (Maestri et al., 2015; Marchand et al., 2003; Oleksyk et al., 2004).

We digitized mandibles from digital photographs taken with a Panasonic Lumix DMC‐FZ47 camera positioned with a tripod and with the hemimandible laid on a surface and supported by the third lower molar and the angular process, so that the view to be photographed (i.e., the external left or right lateral side), was parallel to the camera lens. We centered all specimens to avoid image distortions and did not use zoom. We photographed each hemimandible two times to calculate positioning error (as in Klingenberg, 2015).

**FIGURE 3:**
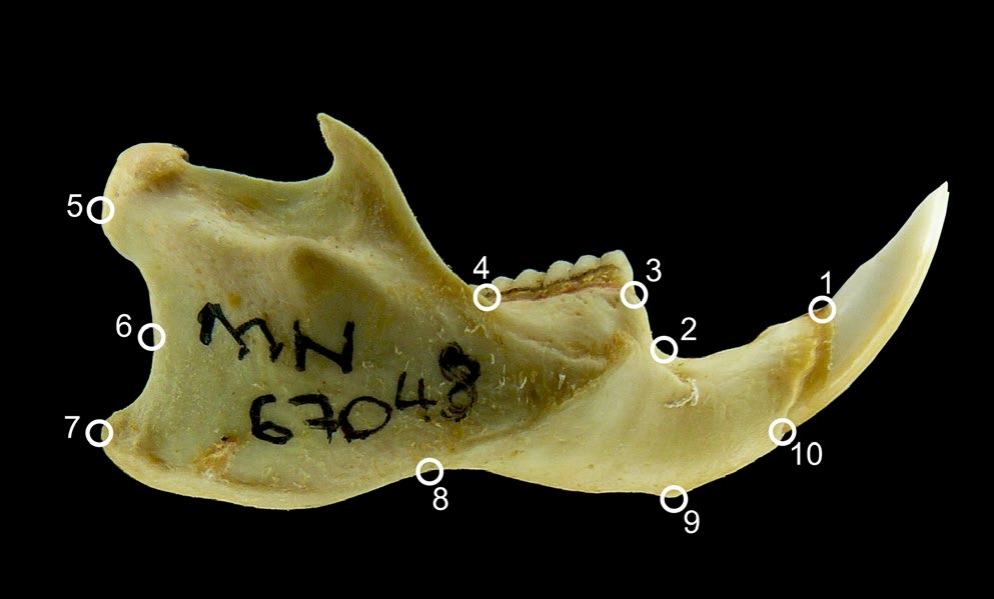
Lateral view of a *Nectomys squamipes* mandible, showing the 10 landmarks used in this work. 1) Anterior edge of the incisive alveolus; 2) Most posterior point of the diastema; 3) Junction between the mandible and the m1 root; 4) Junction between the molar base and the coronoid process; 5) Most posterior point at the articular process; 6) Most anterior point in the angular notch; 7) Most posterior point at the angular process; 8) Angular process base; 9) Inner edge of the mandibular symphysis; 10) Posterior edge of the incisive alveolus

For each photograph, we input 10 anatomical landmarks (Figure 3) using software TPSDIG2 (Rohlf, 2006). We calculated measurement errors in anatomical landmarks inputting landmarks three times for each image and 12 times for each individual (3 inputs for landmarks in 2 mandible sides and 2 pictures each).

We overlapped landmark configurations of all individuals using the Procrustes method (Klingenberg & McIntyre, 1998). We analyzed the presence of FA for mandible shape with a Procrustes ANOVA (Klingenberg & McIntyre, 1998) and the presence of FA for size with a One‐Way ANOVA. For both analyses, we used landmark inputs as error I and images as error II. Following the recommendations of Klingenberg (2015), we classified presence of FA when the effect between the factors “individual” and “side” had a *p* < .05 and the *F* value of this interaction was more than 10 times greater than the *F* value for the error.

For FA in mandible shape and size, we calculated individual indexes and used them for comparisons of FA levels among populations. For shape, we used Mahalanobis FA score generated from Procrustes ANOVA (see Klingenberg & Monteiro, 2005). For size, the generated FA index is univariate, based on centroid size of landmarks configuration, and shows positive and negative values depending on which side is larger (Palmer & Strobeck, 1986, 2003). To improve the comparisons among samples, we used the absolute values, considering deviations of the symmetric configuration independent of direction. We used the software MORPHO J (Klingenberg, 2008) for all treatments of geometric morphometric data, including Procrustes ANOVA, one‐way ANOVA and calculation of FA indexes.

### 2.4 Assessing genetic variation

We extracted DNA from liver tissue samples of specimens using a Qiagen DNeasy tissue mini kit (Qiagen) and used seven polymorphic microsatellite loci following Almeida et al. (2000) and Maroja et al. (2003). Genotyping was carried out at Cornell Life Sciences Core Laboratory Center on an ABI 4,200 sequencer (Applied Biosystems) using the GeneScan 500 LIZ Size Standard (Applied Biosystems). We used Geneious 9.1.8 (Biomatters) to score peaks and assign genotypes. In Arlequin 3.5 software (Excoffier & Lischer, 2010), we tested linkage disequilibrium for all pairs of loci using 10.000 permutations (Lewontin & Kojima, 1960; Slatkin, 1994; Slatkin & Excoffier, 1996) as well as Hardy‐Weinberg equilibrium according to Levene (1949) and to Guo and Thompson (1992), using 1,000 interactions. We used observed heterozygosity (H_o_) per individual and population as the genetic variation index.

We collected genotypic data for 35 individuals that were also measured for FA from four localities: PNRJ Macaé (*n* = 7), PNRJ Carapebus (*n* = 6), Cabiúnas (*n* = 10), and PNMF Atalaia (*n* = 12) localities (Table 1) and obtained population level data from ReBio União (*n* = 8) and Vale do Pamparrão (*n* = 18) from Almeida et al. (2005). We used Genepop (Raymond & Rousset, 1995; Rousset, 2008) to calculate observed heterozygosity and F_is_ as a proxy for inbreeding.

### 2.5 Comparing FA among different sites

We tested the effect of the populations on the FA of *Nectomys squamipes*, using one‐way ANOVA (for normally distributed data) or Kruskal–Wallis ANOVA for non‐parametric data, using Statistica v. 8.0 (Statsoft, 2007), with significance level of *p* < .05.

### 2.6 Habitat amount and FA

We analyzed the relationship between habitat amount and population FA, based on mean mandible FA for both shape and size, using Pearson correlations. In addition to population FA, we also tested the correlation between individual mandible shape/size FA and habitat amount.

### 2.7 Assessing relevance of different factors to FA

We tested whether individual levels of FA were associated with (a) habitat amount and/or other biological factors such as (b) sex, (c) size, and (d) genetic variation. We also tested whether population levels of FA could be explained by (a) habitat amount, (b) population genetic variability (H_o_), and (c) population inbreeding (F_is_). In both cases, we evaluated the relevance of each factor (predictive variable) to FA levels (response variable) by comparing a general linear model containing the predictive factors of interest against an intercept‐only model (null model). Owing to the limited sample sizes, all models were bivariate, with FA values as the normally distributed response variable and the factor of interest as the predictor variable. We used likelihood‐ratio tests (LRT) to assess the goodness of fit of the null and candidate models. To assess statistical significance, we used Bonferroni corrected alfa values for individual (*α* = .01) and populational (*α* = .0125) analyses.

For Pearson correlations and the LRT analysis, we used R v.3.6 (R Core Team, 2014). Finally, we generated Kernel Density plots for mandible size and shape FA using the package ggplotgui (Stulp, 2019) in r.

## 3 RESULTS

### 3.1 Fluctuating asymmetry in *Nectomys squamipes*

Both Procrustes ANOVA and one‐way ANOVA indicated significant variation for the “individual” effect, suggesting that FA in mandibles shape and size exhibit interindividual variation (Table 4). Procustes ANOVA also indicated significant variation for “side,” suggesting that Directional Asymmetry is present in mandibles shape.

**TABLE 4:** Procrustes ANOVA for FA in *Nectomys squamipes* mandible size (left) and shape (right). SS: sum of squares; *df*: degrees of freedom; Ind: Individual. Significant values are in bold

Effect	Size				Shape			
	SS	*df*	*F*	*p*	SS	*df*	*F*	*p*
Individual	5,277.58	85	632.58	<.**0001**	1.02	1,360	10.27	<.**0001**
Side	0.06	1	0.59	.45	0.01	16	6.55	<.**0001**
Ind. × Side	8.34	85	22.71	<.**0001**	0.10	1,360	8.89	<.**0001**
Error 1	1.49	344	0.02	1	0.04	5,504	0.63	1
Residual	141.11	519			0.11	8,304		

The interaction “individual × side”, indicative of FA, was significant for both Procrustes ANOVA and one‐way ANOVA. The *F* values for the factor “individual × side” were 10 times greater than that observed for Error (Table 4), suggesting that the FA levels in mandibles are substantial.

### 3.2 FA variation in *Nectomys squamipes*

**FIGURE 4:**
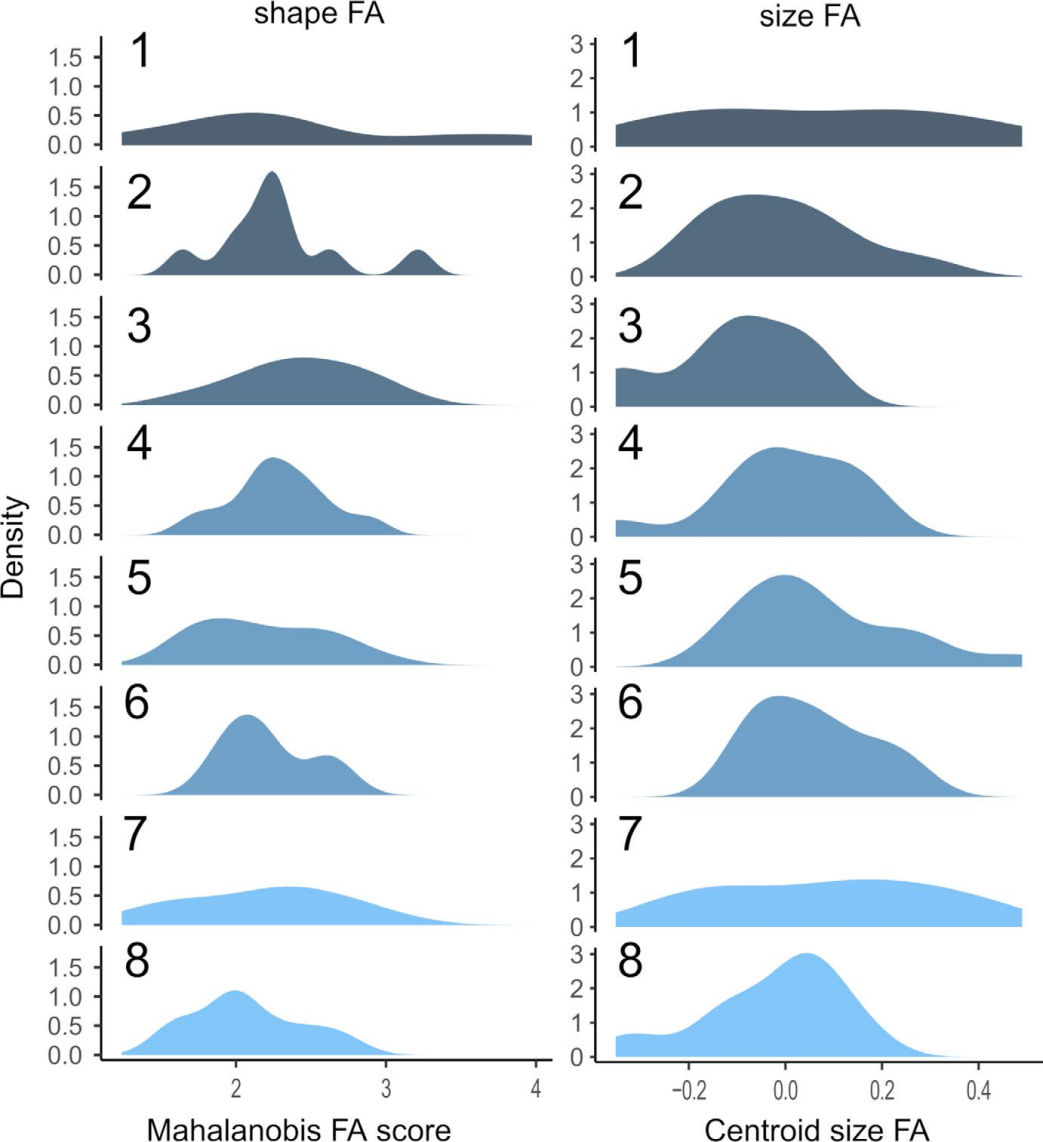
Density distribution of fluctuating asymmetry of mandible shape (Mahalanobis FA score) and size (Centroid size FA) scores in eight populations of *Nectomys squamipes*. Sites: 1—Barra de Maricá; 2—Fazenda Rosimary; 3—PNRJ Lagomar (Macaé); 4—PNRJ São Lázaro (Carapebus); 5—Vale do Pamparrão; 6—Cabiúnas; 7—PNMF Atalaia; 8—ReBio União. Darker colors represent lower habitat amount whereas lighter colors represent higher habitat amount. Habitat amount shown as a percentage in a radius of 264 m of the collection site

Populations presented similar variances for size FA values, with means varying between 0.10 and 0.13, except for samples from PNMF Atalaia (mean = 0.2) and Barra de Maricá (means = 0.23), that had higher means (Table 5). On the other hand, means of FA values related to mandible shape varied gradually between 2.07 (ReBio União) and 2.39 (Barra de Maricá) (Table 5). Differences in FA among populations were not significant (Figure 4) for either size (size [KW‐H (7.86) = 13.78; *p* = .05] or shape [*F* (7.78) = 0.60; *p* = .75]). However, populations did differ on FA shape variance. While most populations presented variances between 0.11 and 0.27, Cabiúnas exhibited a variance of 0.08 while Barra de Maricá a variance of 0.78, a value at least twice as large as the other populations (Table 5).

**TABLE 5:** Descriptive statistics (mean ± σ^2^ [min–max]) of FA indexes for mandible size (module of centroid size) and shape (Mahalanobis FA score) for the eight populations of *Nectomys squamipes,* along with other populational parameters: observed heterozygosity (H_o_), inbreeding coefficient (F_is_), size ratio (given by the percentage of small individuals), and sex ratio (given by the percentage of males). σ^2^ = variance. The % of habitat is the one calculated with the smallest scale effect radius (264 m)

Population (%Habitat)	H_o_	F_is_	Size ratio	Sex ratio	Size FA	Shape FA
1 – Barra de Maricá (18.86%)	—	—	62.5%	50.0%	0.23 ± 0.02 [0.03–0.49]	2.39 ± 0.78 [1.24–3.97]
2 – Fazenda Rosimary (28.15%)	—	—	55.55%	66.7%	0.12 ± 0.01 [0.02–0.28]	2.28 ± 0.19 [1.64–3.21]
3 – PNRJ Macaé (39.26%)	0.84	0.03	30.0%	50.0%	0.13 ± 0.01 [0.04–0.35]	2.37 ± 0.20 [1.68–3.07]
4 – PNRJ Carapebus (66.25%)	0.83	0.09	36.4%	63.6%	0.11 ± 0.01 [0.00–0.35]	2.29 ± 0.11 [1.72–2.89]
5 – Vale do Pamparrão (71.02%)	0.82	0.02	46.7%	73.3%	0.13 ± 0.02 [0.01–0.48]	2.18 ± 0.17 [1.62–2.95]
6 – Cabiúnas (73.22%)	0.70	0.12	63,6%	72.7%	0.10 ± 0.01 [0.03–0.25]	2.21 ± 0.08 [1.85–2.67]
7 – PNMF Atalaia (99.59%)	0.83	0.00	30.8%	61.5%	0.20 ± 0.01 [0.05–0.44]	2.18 ± 0.27 [1.33–3.02]
8 – ReBio União (100%)	0.77	0.06	88.9%	66.7%	0.11 ± 0.01 [0.03–0.32]	2.07 ± 0.14 [1.58–2.71]

### 3.3 Habitat amount and FA

**FIGURE 5:**
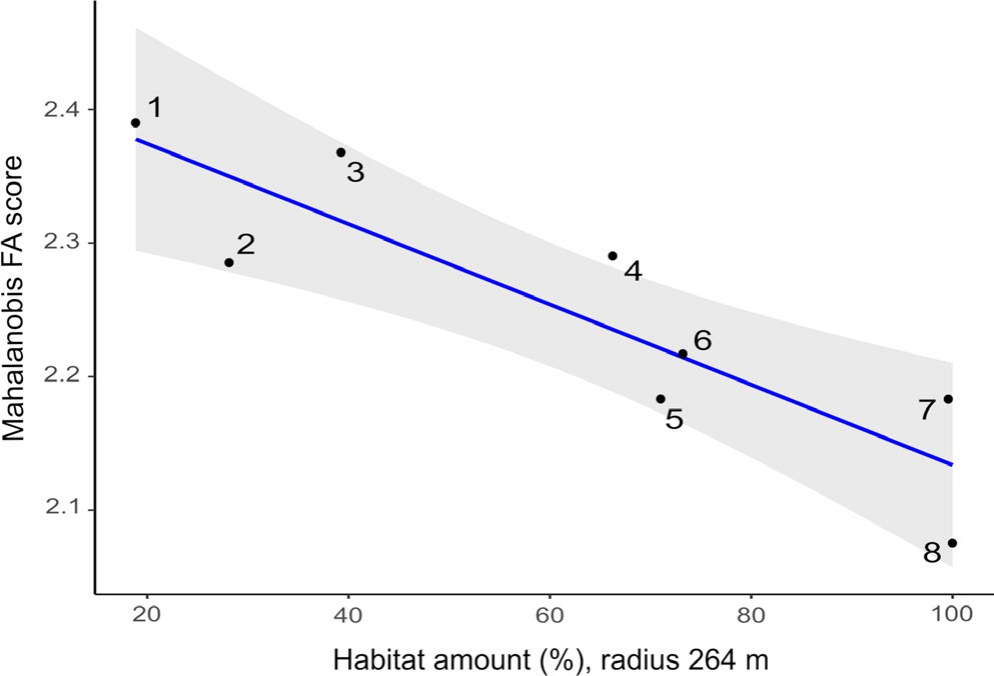
Relationship between habitat amount (measured as percentage in a radius of 264 m of the collection site) and fluctuating asymmetry (Mahalanobis FA score) of mandible shape in eight populations of *Nectomys squamipes*. Regression line shown in blue and confidence interval in grey. Sites: 1—Barra de Maricá; 2—Fazenda Rosimary;3—PNRJ Lagomar (Macaé); 4—PNRJ São Lázaro (Carapebus);5—Vale do Pamparrão; 6—Cabiúnas; 7—PNMF Atalaia; 8—ReBio União

We tested the relationship between FA, both in size and shape, and habitat amount. We used mean FA values and percentiles of habitat available in each site calculated using scale effect values (Table 3). Only percentages calculated using a radius of 264m showed a significant correlation with FA values. Populational FA indexes for mandible size were not correlated with habitat amount (*r* = −.22; *p* = .59; Figure 4). However, FA indexes for mandible shape exhibited significant and strong negative correlation (*r* = −.88; *p* = .00) with habitat amount percentiles, suggesting that, in sites with more habitat available, such as PNMF Atalaia and ReBio União, mean FA values were lower than the observed in sites with less habitat available, such as in Barra de Maricá (Figure 5). Considering individual FA scores, no correlation was observed between size (*r* = −.04; *p* = .72) or shape FA (*r* = −.19; *p* = .08) and habitat availability.

LRT (Table 6) for individual levels suggested that shape FA levels in *Nectomys squamipes* samples could not be explained by any biological factor (i.e., sex, body size, or heterozygosity). For size FA levels, most biological factors were also not significant. Heterozygosity (Table 6) was not significant for either FA shape or size, suggesting that neither individual nor populational FA levels are associated with genetic diversity. For populational FA in size (Table 6), neither habitat amount, population heterozygosity, nor inbreeding were significant. In contrast, habitat availability was significantly associated with shape FA levels of populations (*p* = .00), suggesting that higher habitat amount is associated with a reduced populational FA in shape.

**TABLE 6:** Likelihood‐ratio tests comparing different factors with a null model as an explanation for individual (Ind.) and populational (Pop.) FA levels in *Nectomys squamipes* populations. Bold values represent significant p values (*p* < .01 for individual analysis and <.0125 for populational analysis)

Factors	Shape FA		Size FA	
	*χ* ^2^	*p* value	*χ* ^2^	*p* value
Ind – habitat amount	3.15	.07	0.13	.77
Ind – sex	1.70	.43	0.29	.86
Ind – body size	0.88	.35	5.66	.02
Ind – H_O_	0.08	.77	2.60	.10
Pop – Habitat amount	12.00	.**00**	0.41	.52
Pop – H_O_	0.83	.36	1.90	.17
Pop – F_is_	0.01	.92	5.36	.02

## 4 DISCUSSION

Here, we show that the substantial Fluctuating Asymmetry (FA) in *Nectomys squamipes* mandibles is associated with habitat amount, at least regarding mandible shape FA at the population level. Apart from habitat amount, no other biological factors (sex, size, genetic variation, and inbreeding) were associated with mandible shape FA. These results suggest that although *N. squamipes* is still present in landscapes with low forest cover, its populations might already show the phenotypic effects of environmental stressors, which precede declines in local adaptive values. Therefore, FA measures from scientific collections might be promising as a method to monitor populations under various environmental conditions and through time, helping biodiversity conservation efforts (Williams, 2000).

### 4.1 Detection of FA levels and its relationships with habitat amount and genetic variation

In FA studies, comparisons among populations are based on differences in their variances. Therefore, these studies depend on an accurate measure of variance and, consequently, on the number of samples (Graham et al., 2010). Because our sample size is limited, our power to detect differences between individuals and populations might be low (see Palmer & Strobeck, 1986 , 2003). However, despite this limitation, we did detect an association between FA variances and habitat amount. For mandible shape, seven of the eight populations presented similar variances, while the population of Barra de Maricá presented the highest variance, three times greater than the second highest variance. This locality has the lowest habitat amount, only 18%, while the other localities present more than 28% habitat amount. It is possible that *N. squamipes* exhibits a certain developmental tolerance up to a certain habitat amount threshold, below which more individuals would depart from the mean FA levels. Landscape ecology studies have demonstrated that community integrity of Atlantic forest vertebrates is generally preserved until 24%–33% (~30%) of forest cover, beyond which further habitat loss causes sharp declines in species richness and abrupt increases in local extinction risks for forest specialists (Banks‐Leite et al., 2014; Estavillo et al., 2013; Pardini et al., 2010). Therefore, as a forest‐dependent species, *N. squamipes* might face more stressful conditions in landscapes below 30% of forest cover, signaling higher populational levels of FA, as observed in the Barra de Maricá population.

Landscapes produced by deforestation usually include an extensive matrix of anthropogenic habitats, such as roads, cropland, urban, and/or industrial areas. In these landscapes, populations of forest‐dwelling species are more vulnerable to a number of environmental threats (Graham et al., 2010), such as invasive species (Doherty et al., 2017), pathogens (Smith et al., 2009; Tompkins et al., 2011), and pollutants (Gall et al., 2015). Decreased habitat amount might also lead to increased local population densities resulting in more resource competition, territorial disputes, predation pressure, and conditions that might affect developmental stability and increase FA levels (Badyaev et al., 2000; Møller & Swaddle, 1997). Furthermore, degraded Atlantic forest remnants facilitate exposure of wildlife to domestic animals increasing parasitic infections, such as schistosomiasis. This introduced human parasite is often present in *N. squamipes* populations living in small forest patches near rural and peri‐urban areas (Gentile et al., 2006). Parasite infection is a known stress factor that has been shown to increase fluctuating asymmetry levels (Barnard et al., 2002; Møller & Swaddle, 1997).

Some studies reporting relationships between FA levels and habitat availability or quality presume that the increase in developmental instability is caused by loss of genetic variation in populations (e.g. Anciães & Marini, 2000). This assumption is based on the expectation that inbreeding reduces canalization and increases developmental instability, but the evidence linking FA to heterozygosity is weak (Britten, 1996; Pertoldi et al., 2006; Vøllestad et al., 1999). We did not detect relationships between populational or individual level FA with observed heterozygosity or F_is_, suggesting that loss of genetic variation or inbreeding did not explain the occurrence of highly asymmetric individuals in populations. The heterozygosity levels observed were generally high (as in other studies, see Almeida et al., 2005), suggesting that the populations studied did not experience severe loss of genetic diversity. The relationship between genetic variation and FA levels might be non‐linear and difficult to detect in populations maintaining high genetic diversity. White and Searle (2008), for instance, reported that lack of habitat becomes relevant only in very small populations suffering from inbreeding depression. In a survey of island populations of the common shrew (*Sorex araneus*), White and Searle (2008) recovered a positive correlation between FA and genetic diversity. However, this relationship was driven by a single small island population exhibiting both the highest FA and lowest expected heterozygosity. It is possible that, if we had genetic data for the population of Barra de Maricá, our results would uncover a similar relationship. Further studies including more samples from low‐diversity (and/or small habitat amount) populations are needed to test the hypothesis of genetic diversity as a cause of FA.

It is interesting that, in contrast to the patterns of populational FA levels, individual FA levels were not related to habitat amount or to genetic variation, suggesting that they are best explained by chance. The lack of correlations was due to the pervasive occurrence of symmetrical individuals in populations with low habitat amount, and of asymmetrical individuals with high heterozygosity. The first case is clearly illustrated by the population of Barra de Maricá, which exhibits very symmetrical individuals together with the most asymmetrical ones, increasing the FA mean and variance of the population. In the second case, it could be argued that the microsatellite loci variation assessed by us do not adequately represents genome wide diversity (Zachos et al., 2007). If FA is associated to variation in a few key loci not included in the genotyping (Vangestel et al., 2011), it would not be possible to detect correlations between heterozygosity and individual FA. Despite these uncertainties, our results suggest that FA might be more appropriate as a populational rather than individual indicator of stress and that further research is needed on the relationship between FA and heterozygosity.

### 4.2 Fluctuating asymmetry and conservation

Several studies have suggested the use of Fluctuating Asymmetry as a potential indicator for environmental stresses (e.g., Leamy et al., 1999; Marchand et al., 2003; Oleksyk et al., 2004), including habitat loss and fragmentation (Anciães & Marini, 2000; Wauters et al., 1996). The ability to detect stress prior to its more severe consequences, such as changes in the adaptive value, presence of large deformations and severe population size declines (Sarre & Dearn, 1991), suggest that FA studies might be used for monitoring endangered species (Schmeller et al., 2011). Furthermore, while our study employed methods only suitable to museum specimens, several studies using live individuals have also reported a positive relationship between environmental stress and the increase of FA levels in small mammal populations (Coda et al., 2016; Hopton et al., 2009; Wauters et al., 1996).

The present study is among the few to use Fluctuating Asymmetry levels as indicators of environmental stress for small Neotropical mammals. In addition, it is one of the first to investigate such relationships for sigmodontine rodents in remnants of Atlantic forest using museum collections to access the consequences of anthropogenic and environmental impacts in natural populations (Askay et al., 2014; Maestri et al., 2015). Despite the small sample sizes inherent in studies with mammals, we were able to show a relationship between habitat amount and the magnitude of mandible asymmetry, providing support for the use of this methodology as indicator of environmental stress caused by habitat restriction in Neotropical small mammals.

## CONFLICT OF INTERESTS

None declared.

## AUTHOR CONTRIBUTION

**Aldo Caccavo:** Conceptualization (equal); Formal analysis (lead); Investigation (equal); Visualization (equal); Writing‐original draft (lead); Writing‐review & editing (equal). **Hudson Lemos:** Formal analysis (equal); Investigation (equal); Visualization (supporting); Writing‐review & editing (equal). **Luana S.** **Maroja** **:** Investigation (supporting); Supervision (supporting); Visualization (supporting); Writing‐review & editing (equal). **Pablo Rodrigues Gonçalves:** Conceptualization (equal); Funding acquisition (lead); Investigation (supporting); Supervision (lead); Writing‐original draft (supporting); Writing‐review & editing (equal).

## Supporting information

Supplementary Material

## Data Availability

All data used in this paper have been deposited in Dryad accession number: https://doi.org/10.5061/dryad.dz08kprwq
