# Supplementary material for: Does stress mess with rodents’ heads? Influence of habitat amount and genetic factors in mandible fluctuating asymmetry in South American water rats (Nectomys squamipes, Sigmodontinae) from Brazilian Atlantic rainforest remnants
Source: Ecol Evol. 2021 May 2;11(11):7080–92. doi: 10.1002/ece3.7557 (PMC8207160; doi:10.1002/ece3.7557)
Supplement: Supplementary file 1 — Supplementary Material [file ECE3-11-7080-s001.docx]

# Appendix

The specimens used in the present work are listed below. Each individual is identi.fied using the catalog number of the mammal collection of Museu Nacional (MN) or NUPEM/UFRJ (NPM). In addition, the sex are identified with ‘♂’ for males, ‘♀’ for females, and ‘?’ for unknown. Specimens with genetic data available are marked with “*”.

Cachoeiras de Macacu – Fazenda Rosimary 22°29'S 42°51'W: MN67044 ♂; MN67045 ♂; MN67046 ♀; MN67047 ♂; MN67048 ♀; MN67051 ♂; MN67052 ♀; MN67053 ♂; MN67054 ♂.

Carapebus – Parque Nacional da Restinga de Jurubatiba, São Lázaro 22°18'54.32''S, 41°59'37.48''W: NPM089 ♀; NPM098* ♂; NPM099* ♂; NPM115* ♀; NPM116* ♂; NPM117* ♂; NPM366 ?; NPM446 ♀; NPM641 ♂; NPM924 ♂.

Casimiro de Abreu – Reserva Biológica União 22°25'35''S, 41°43'40.94''W: MN33796 ♂; MN33797 ♀; MN33799 ♂; MN33800 ♂; MN33801 ♂; MN33803 ♀; MN33804 ♂; MN33805 ♂; MN74390 ♀.

Macaé – Cabiúnas 22°17'28.51 ''S, 41°59'37.48''W: NPM076* ♂; NPM086 ♂; NPM088* ♂; NPM147* ♀; NPM285* ♀; NPM313* ♂; NPM324* ♂; NPM326 ♂; NPM327* ♂; NPM369* ♀; NPM420* M. Parque Na23cional da Restinga de Jurubatiba, Lagomar 22°17'25.11''S, 41° 41'55.64''W: NPM632 ♂; NPM823 ♂; NPM840 ♀; NPM991 ♀; NPM994* ♂; NPM995* ♀; NPM996* ♀; NPM1136* ♂; NPM1137* ♂; NPM1141* ♀. Parque Natural Municipal Fazenda Atalaia 22°18'54.32''S, 41°59'37.48''W: NPM013* ♀; NPM024* ♀; NPM026* ♂; NPM040* ♂; NPM043* ♀; NPM048* ♀; NPM049* ♂; NPM050* ♀; NPM239* ♂; NPM241* ♂; NPM733 ♂; NPM747 ♂; NPM1166* ♂.

Maricá – Barra de Maricá 22°57'S 42°50'W: MN27830 ♀; MN27832 ♂; MN28540 ♀; MN28541 ♀; MN28545 ♂; MN28546 ♂; MN28549 ?; MN28601 ♂.

Sumidouro– Vale do Pamparrão 22°02'46''S 42°41'21''W: MN26840 ♂; MN27102 ♂; MN50510 ♂; MN50515 ♂; MN50520 ♂; MN50529 ♀; MN50530 ♂; MN50531 ♂; MN50532 ♀; MN50533 ♀; MN50538 ♂; MN50540 ♀; MN50541 ♂; MN50544 ♂; MN50561 ♂.
